# The US Opioid Epidemic and Its Impact on US General Practice Veterinarians

**DOI:** 10.3389/fvets.2019.00222

**Published:** 2019-07-04

**Authors:** Lori Kogan, Peter Hellyer, Mark Rishniw, Regina Schoenfeld-Tacher

**Affiliations:** ^1^Department of Clinical Sciences, College of Veterinary Medicine and Biomedical Sciences, Colorado State University, Fort Collins, CO, United States; ^2^Veterinary Information Network, Davis, CA, United States; ^3^Department of Molecular Biomedical Sciences, College of Veterinary Medicine, North Carolina State University, Raleigh, NC, United States

**Keywords:** opioid, veterinary, shortage, pain management, anesthesia

## Abstract

**Objective:** To assess the impact of the human opioid epidemic and associated shortages in drug supply on US general practice veterinarians.

**Design:** Cross-sectional study.

**Sample:** Members of the Veterinary Information Network (VIN).

**Procedures:** An electronic survey was used to examine veterinarians' views regarding opioid use in veterinary medicine and the impact of the opioid shortage on the provision of care. The survey was distributed via the VIN data collection portal from October 12–November 6, 2018.

**Results:** 697 veterinarians completed the survey. Most (99.7%) reported using, dispensing or prescribing opioids in veterinary practice. The most commonly used opioids were buprenorphine, tramadol and butorphanol. While most veterinarians (83.3%) reported difficulty in ordering opioids over the last 6 months, this decreased to 59.0% in the last month. The most difficult drugs to obtain were hydromorphone, morphine, injectable fentanyl, and oxymorphone. The reported rate of difficulty in obtaining all these drugs lessened over time. However, the opioid shortage caused significant difficulty in providing appropriate pain management for 41.1% of participants, and affected the ability of 44.8% of respondents to provide optimal anesthesia.

**Conclusions and Clinical Relevance:** Veterinarians' ability to provide opioids for their patients has been impacted by the opioid shortage, with a greater impact on full mu opioid agonists as compared to drugs like butorphanol, buprenorphine, and tramadol. The results confirm the important role of opioid analgesics in the delivery of modern veterinary medicine and highlight the importance of medical health professionals being able to access these critical medications.

## Introduction

Anecdotal evidence suggests that veterinarians have been struggling to obtain sufficient quantities of opioid analgesics for their patients. The apparent shortage of opioids may be dues to multiple factors, such as manufacturing shortages and changes in compounding regulations; however, the opioid epidemic stands out as a significant contributing factor. According to the National Institute on Drug Abuse (https://www.drugabuse.gov/drugs-abuse/opioids/opioid-overdose-crisis), the Opioid Overdose Crisis (also known as the opioid crisis) has the following characteristics as of January 2019: “Every day, more than 130 people in the United States die after overdosing on opioids (1). The misuse of and addiction to opioids—including prescription pain relievers, heroin, and synthetic opioids such as fentanyl—is a serious national crisis that affects public health as well as social and economic welfare. The Centers for Disease Control and Prevention estimates that the total ‘economic burden' of prescription opioid misuse alone in the United States is $78.5 billion a year, including the costs of healthcare, lost productivity, addiction treatment, and criminal justice involvement (2).”

In order to understand the impact of the opioid crisis on veterinary medicine it is vital to understand the role that human medicine and the well-intentioned desire to control pain in people contributed to where we are at now. The United States is in the midst of an opioid addiction epidemic (1–8) while simultaneously experiencing a shortage of opioids for use in legitimate medical situations, including the delivery of veterinary medicine. A wide variety of opioids are used in veterinary medicine to provide pain relief and as part of a balanced anesthestic protocol, including butorphanol, buprenorphine, fentanyl, hydromorphone, methadone, and morphine (9). There are numerous anecdotal stories of pet owners seeking opioids from veterinarians for their own use as well as owners requesting opioids for their pets when they are not needed or recommended (10). In response to this crisis, many states are passing laws to help monitor opioid prescriptions. In several states, professionals (including veterinarians) are now required to check the prescription drug monitoring program (PMP, a statewide database) and potentially notify authorities if a client meets specific criteria. Other states have begun limiting opioid prescriptions to 5–7 days (11).

A recent survey of 189 Colorado veterinarians found that 40% of respondents were unsure if there was an opioid problem in their community, but 62% felt that they have a role in preventing opioid misuse. Additionally, 44% of respondents were aware of opioid abuse or misuse by either a client or staff member and 13% were aware of a client who had intentionally made an animal ill or injured (or appear ill or injured) to obtain opioid medications (12). The researchers suggested that additional resources should be made available for tracking and surveillance, education, and further research.

In an effort to help support veterinarians, FDA commissioner Scott Gottlieb published a statement in August 2018 offering a resource to help veterinarians responsibly prescribe opioids (13). The resource includes suggestions to follow all state and federal regulations when ordering and prescribing opioids, use non-opioids when possible, educate pet owners on the risks of opioids, and educate oneself about opioid abuse—including signs/symptoms and a plan about how to proceed if they encounter opioid diversion or abuse (14). Additional opioid—related resources for veterinarians, created by the AVMA, include a list of states' PMPs and continuing education related to opioids, a resource to help veterinarians identify “vet shopping” and drug diversion, and the AVMA's policy on the veterinarian's role in addressing the opioid epidemic (15).

These resources and guidelines were created because there are times when opioids are the best medical choice and ensuring that veterinarians are able to provide these medications to their patients is critically important. Paradoxically, in the presence of an opioid crisis anecdotal stories suggest that veterinarians are struggling to obtain opioids for their patients. This current study is the first one designed to assess how the opioid crisis has affected general practice veterinarians and their ability to provide optimal treatment to their clients.

## Materials and Methods

An anonymous online survey was created, in collaboration with VIN (an online veterinary community), to evaluate veterinarians' views regarding opioids and the impact of the opioid shortage on the practice of veterinary medicine. The survey was created and tested by researchers at Colorado State University and VIN. After the survey was created, an online distribution was arranged for a small sample of VIN members in order to pilot the survey. They assessed the survey for appropriate branching and question flow, ambiguity, and potentially missing or inappropriate response options. Their feedback was analyzed, and incorporated into the final version of the survey. A link to the survey was distributed via an email invitation to all VIN members (*n* ~ 35,000), and access was made available from October 12–November 6, 2018. US VIN members practice in all 50 US states, have an average age of 45.5 and are approximately 69% female. The authors believe that VIN membership is reflective of the veterinary profession at large. A follow-up message was sent 2 weeks after the initial invitation. Only data from respondents who stated they currently practice general veterinary medicine in the US were included in the study. The study was categorized as exempt by Colorado State University's Institutional Review Board. Because this was an anonymous survey, written informed consent was not required. An introductory statement explained the study and indicated to potential participants that consent was implied by completing the survey.

The survey was administered directly via the VIN data collection portal, and branching logic was used to display only questions relevant to each participant. The first two questions were screening tools to ensure respondents were general practitioners practicing in the US. Veterinarians who self-identified as not in general practice (e.g., specialists) or did not practice in the US were eliminated from further analysis. The body of the survey consisted primarily of short questions, for which participants were able to select one or more specific options to represent their experiences and perceptions regarding opioid use, prescribing and dispensing. Free-text boxes were provided for participants to enter brief alternative answers when none of the listed options applied to them. A final question at the end of the survey allowed for free-text entry of any comments participants chose to make about the current opioid shortage.

## Results

### Opioid Use

The number of respondents who were general practitioners practicing in the US, and therefore eligible for analysis, was 697. Nearly all respondents indicated they use, dispense or prescribe opioids (695/697; 99.7%). Participants were asked to indicate in what ways they work with opioids (prescribe, dispense, or use in clinic). The most common response was use in clinic (682, 97.8%), followed by dispense to clients (560, 80.3%), and prescribe to outside pharmacy (490, 70.3%).

Participants were next asked to indicate any/all of the opioids they use, prescribe or dispense from a list of the most common opioids. The most commonly used opioids were Buprenorphine, Tramadol, and Butorphanol (Table 1).

**Table 1:** Reported usage of common opioids by 697 veterinarians.

**Opioid**	**Reported number of veterinarians using in practice**
Buprenorphine	669 (96.0%)
Tramadol	633 (90.8%)
Butorphanol	631 (90.5%)
Hydromorphone	500 (71.7%)
Hydrocodone	428 (61.4%)
Morphine	310 (44.5%)
Fentanyl patch	190 (27.3%)
Fentanyl injectable	149 (21.4%)
Methadone	76 (10.9%)
Oxymorphone	39 (5.6%)

For each opioid, participants were asked to specify how they interfaced with the drug: via use in clinic, prescribing to patients or dispensing to clients (or any combination of the three). For all drugs participants reported using in one of these three ways, they were asked to indicate how frequently they prescribed (Table 2) used in the clinic (Table 3) and dispensed (Table 4).

**Table 2:** Veterinarians' reported frequency of prescribing specific opioids in veterinary practice.

**Opioid**	**Prescribe frequently**	**Prescribe infrequently**	**Don't prescribe**
Tramadol (*n* = 458)	245 (53.5%)	139 (30.3%)	74 (16.2%)
Buprenorphine (*n* = 475)	126 (26.5%)	74 (15.6%)	275 (57.9%)
Hydrocodone (*n* = 342)	98 (28.7%)	197 (57.6%)	47 (13.7%)
Fentanyl patch (*n* = 141)	9 (6.4%)	55 (39.0%)	77 (54.6%)
Butorphanol (*n* = 439)	22 (5.0%)	62 (14.1%)	355 (80.9%)
Methadone (*n* = 51)	2 (3.9%)	3 (5.9%)	46 (90.2%)
Fentanyl injectable (*n* = 95)	3 (3.2%)	4 (4.2%)	88 (92.6%)
Hydromorphone (*n* = 351)	10 (2.8%)	12 (3.4%)	329 (93.7%)
Morphine (*n* = 214)	4 (1.9%)	7 (3.3%)	203 (94.9%)
Oxymorphone (*n* = 27)	-	2 (7.4%)	25 (92.6%)

**Table 3:** Veterinarians' reported frequency rates of administration of specific opioids in a clinic setting.

**Opioid**	**Administer frequently**	**Administer infrequently**	**Don't administer**
Buprenorphine (*n* = 659)	581 (88.2%)	70 (10.6%)	8 (1.2%)
Butorphanol (*n* = 624)	492 (78.8%)	115 (18.4%)	17 (2.7%)
Hydromorphone (*n* = 495)	385 (77.8%)	99 (20.0%)	11 (2.2%)
Methadone (*n* = 75)	48 (64.0%)	25 (33.3%)	2 (2.7%)
Morphine (*n* = 308)	184 (59.7%)	118 (38.3%)	6 (1.9%)
Fentanyl injectable (*n* = 147)	74 (50.3%)	70 (47.6%)	3 (2.0%)
Tramadol (*n* = 623)	270 (43.3%)	122 (19.6%)	231 (37.1%)
Fentanyl patch (*n* = 187)	47 (25.1%)	123 (65.8%)	17 (9.1%)
Hydrocodone (*n* = 420)	94 (22.4%)	128 (30.5%)	198 (47.1%)
Oxymorphone (*n* = 39)	8 (20.5%)	27 (69.2%)	4 (10.3%)

**Table 4:** Veterinarians' reported frequency rates for dispensing specific opioids in a clinic setting.

**Opioid**	**Dispense frequently**	**Dispense infrequently**	**Don't dispense**
Buprenorphine (*n* = 539)	279 (51.8%)	180 (33.4%)	80 (14.8%)
Tramadol (*n* = 524)	350 (66.8%)	151 (28.8%)	23 (4.4%)
Hydrocodone (*n* = 369)	119 (32.2%)	137 (37.1%)	113 (30.6%)
Fentanyl patch (*n* = 164)	15 (9.1%)	59 (36.0%)	90 (54.9%)
Butorphanol (*n* = 503)	25 (5.0%)	144 (28.6%)	334 (66.4%)
Hydromorphone (*n* = 395)	6 (1.5%)	21 (5.3%)	368 (93.2%)
Methadone (*n* = 70)	1 (1.4%)	7 (10.0%)	62 (88.6%)
Morphine (*n* = 240)	2 (0.8%)	14 (5.8%)	224 (93.3%)
Oxymorphone (*n* = 34)	–	3 (8.8%)	31 (91.2%)
Fentanyl injectable (*n* = 126)	–	5 (4.0%)	121 (96.0%)

Next, participants who reported ordering and using opioids in the clinic were asked if they had experienced difficulties in obtaining opioids for use in the clinic or dispensing to clients in either the last 6 months or last 30 days. Difficulties obtaining opioids in the last 6 months (*n* = 682) was reported by 568, 83.3%. Fewer practitioners (101, 14.8%) reported no difficulties, and 13 (1.9%) said they did not know. Difficulties in the last 30 days (*n* = 669) was reported by 395 (59.0%), with 207 (30.9%) reporting not experiencing recent difficulties, and 67 (10.0%) reporting they did not know.

Similarly, participants who reported prescribing opioids to clients were asked to indicate if they have had challenges in the last 6 months (*n* = 485) or the last 30 days (*n* = 477). Reports of difficulties in the last 6 months were reported by 141 (29.1%); with 322 (66.4%) reporting no difficulties, and 22 (4.5%) saying they do not know. When asked about challenges in the last 30 days, 103 (21.6%) reported challenges, 339 (71.1%) reported no challenges, and 35 (7.3%) said they did not know.

Participants were then asked to report difficulties within the last 6 months and within the last month in obtaining each of the drugs they reported using (Table 5). These responses showed the most difficult drugs to obtain in the last 6 months were Fentanyl injectable, Hydromorphone, Morphine, and Oxymorphone. The reported difficulties obtaining these drugs was less in the past month when compared to challenges in the last 6 months.

**Table 5:** Veterinarians' reported difficulties obtaining specific opioids (only for those who said they use each of these drugs).

Opioid^*^	**Difficulties obtaining in last 6 months**	**Difficulties obtaining in last month**	**No difficulty obtaining**
Hydromorphone (*n* = 500)	408 (81.6%)	184 (36.8%)	53 (10.6%)
Morphine (*n* = 310)	232 (74.8%)	120 (38.7%)	46 (14.8%)
Fentanyl injectable (*n* = 149)	107 (71.8%)	34 (22.8%)	31 (20.8%)
Oxymorphone (*n* = 39)	28 (71.8%)	15 (38.5%)	9 (23.1%)
Butorphanol (*n* = 631)	42 (6.7%)	17 (2.7%)	540 (85.6%)
Buprenorphine (*n* = 669)	178 (26.6%)	46 (6.9%)	435 (65.0%)
Hydrocodone (*n* = 428)	141 (32.9%)	64 (15.0%)	236 (55.1%)
Methadone (*n* = 76)	44 (57.9%)	28 (36.8%)	16 (21.1%)
Tramadol (*n* = 633)	27 (4.3%)	13 (2.1%)	572 (90.4%)
Fentanyl patch (*n* = 190)	47 (24.7%)	14 (7.4%)	128 (67.4%)

* *Numbers in this column reflect the total number of participants who reported using each opioid. Two additional separate questions were asked about difficulties obtaining the opioid (in last 6 months and last month). These questions were not required and therefore not answered by all those who indicated they used the opioid*.

### Impact of Opioid Shortage on Providing Appropriate Pain Management

Participants were asked to rate the impact of the opioid shortage (on a scale from 1—no difficulty to 10—significant difficulty) on being able to provide appropriate pain management to their patients and providing optimal anesthesia that utilizes opioids. These scores were divided into three groups: no/little difficulty (scores 1–3), moderate difficulty (4–7), and significant difficulty (scores 8–10). Participants reported high levels of difficulty in both these areas (Table 6).

**Table 6:** Areas of clinical practice affected by the opioid shortage as reported by practicing veterinarians.

**Opioid shortage**	**No/little difficulty**	**Moderate difficulty**	**Significant difficulty**
Affected ability to provide appropriate pain management (*n* = 694)	175 (25.2%)	234 (33.7%)	285 (41.1%)
Affected ability to provide optimal anesthesia (*n* = 692)	184 (26.6%)	198 (28.6%)	310 (44.8%)

When participants were asked if they have canceled or postponed any procedures due to the opioid shortage in the last 6 months (*n* = 638), 549 (86.1%) said no, 18 (2.8%) said not applied, and 71 (11.1%) said yes. They were then asked if they had discussed the opioid shortage with clients in situations where their pet requires opioid treatment (*n* = 624). A total of 316 (50.6%) said no, 29 (4.6%) said not applicable, and 279 (44.7%) said yes.

Lastly, participants were asked if they were concerned about opioid diversion from their patients to people who work in the hospital (*n* = 691) or the pet's owners (*n* = 694). The number of participants who reported concern for clinic workers was less than the concern about diversion among pet owners. Concern for diversion to people within the clinic was noted by 171 (24.7%) of participants, 485 (70.2%) said they were not concerned about diversion to people within the clinic, 28 (4.1%) said they had not thought about it, and 7 (1.0%) said it was not applicable to them. When asked about concern for diversion to pet owners, 441 (63.5%) said yes they were concerned, 231 (33.3%) said they were not concerned, 13 (1.9%) said they had never thought about it, and 9 (1.3%) said it was not applicable.

## Discussion

Despite the widespread focus on the epidemic of opioid overuse/abuse in humans, there is a concurrent, poorly described opioid shortage for legitimate veterinary clinical use. The reasons for the shortage of clinically used opioids are unclear; however, anecdotally it appears the shortages have made an impact on veterinarians who use opioids in their practice. This is the first survey to the authors' knowledge to explore this issue. Of the 697 VIN members who completed the survey, 99.7% indicated they use, dispense, or prescribe opioids. This result is not surprising considering that opioids are a fundamental part of anesthetic protocols and acute pain management, especially in small animal patients (9, 16, 17). Buprenorphine, tramadol, and butorphanol were the most widely used opioids, reflecting the importance of these drugs in anesthetic, sedation, and acute pain management protocols as well as veterinarian comfort with using these opioids. Interestingly, morphine was used by 44.5% of respondents, probably reflecting morphine's efficacy for moderate to severe pain, good sedative properties, and affordability (9, 16–19).

Of the drugs utilized by survey participants, tramadol was the most commonly prescribed for outpatient use (245 out of 458, 53.5%) followed by buprenorphine (126 out of 475, 26.5%) and hydrocodone (98 out of 342, 28.7%). Although not specifically explored in the survey, it is likely that hydrocodone is prescribed primarily for cough suppression rather than pain control (20). Buprenorphine is often a good choice for postoperative pain control in dogs and cats and has the advantages of long lasting analgesic effect (~8 h) and high bioavailability following trans-mucosal administration (21–24). Tramadol has gained popularity over several years for the treatment of acute and chronic pain in dogs and cats; however, its efficacy has been questioned recently (25, 26). It is possible that some dogs administered tramadol do not experience any significant pain control. The popularity of tramadol may be due in part to the lack of effective and practical alternatives and anecdotal evidence suggesting that it has analgesic properties in some dogs. The dispensing rate from the in- hospital pharmacy (as opposed to prescribing to an outside pharmacy) followed the same pattern for tramadol, buprenorphine, and hydrocodone.

In clinic administration of opioids was most common for butorphanol, buprenorphine, tramadol, and the mu opioid agonists (fentanyl, hydromorphone, methadone, morphine). Oxymorphone, also a mu opioid agonist, was used less frequently than the other mu agonists (8 out of 39, 20.5%), likely due to a lack of widespread availability. It is not surprising that the mu opioid agonists are used primarily in clinic since they are available as injectable drugs and are commonly used in anesthetic protocols, especially for painful procedures. The greater frequency of mu-opioid agonist use in clinic compared to relatively few times these drugs were prescribed or dispensed may be due to concerns over having injectable opioids leave a clinic, lack of efficacy of oral mu agonists in dogs and cats, and veterinarian concern over diversion of potent opioid drugs.

Participants reported having difficulty in ordering opioids for their clinic in the last 6 months (568 out of 682, 83.3%) and in the last 30 days (395 out of 669, 59%) suggesting that the opioid shortage may be improving. When asked about the difficulty in prescribing opioids for their patients, 141 out of 485 (29.1%) had experienced difficulty in the last 6 months, and 103 out of 485 (21.6%) had experienced difficulty in the last 30 days. The difficulty in prescribing opioids mirrored the experience with ordering drugs for in clinic use in that the difficulty seems to have improved in the last 30 days as compared to 6 months ago. Interestingly, it was more difficult for these veterinarians to order opioids for their clinic than it was to prescribe them for their patients, possibly reflecting the greater buying power that many pharmacies have compared to individual in clinic veterinary pharmacies.

For veterinarians experiencing difficulty ordering opioids over the past 6 months, hydromorphone, morphine, injectable fentanyl, oxymorphone, and fentanyl patches were the most difficult to obtain. These results are not surprising considering that all of these drugs are Schedule II drugs indicating a legitimate medical use with a high potential for abuse, with use potentially leading to severe psychological or physical dependence (27). Although only 190 respondents ordered fentanyl patches, 47 (24.7%) experienced difficulty ordering fentanyl in this formulation. Fentanyl patches seem to have lost popularity in veterinary medicine over the last several years as studies have documented inconsistent transdermal absorption (28–31). Anecdotally, veterinarians appear to be concerned about sending patients home with a transdermal fentanyl patch applied due to the potential for diversion of the powerful opioid in the patch.

Participants were asked about the impact of the opioid shortage on their ability to practice veterinary medicine. Specifically, the opioid shortage impacted the veterinarian's ability to provide appropriate pain management significantly (285 out of 694, 41.1%) or moderately (234 out of 694, 33.7%). Similarly, the opioid shortage affected their ability to provide optimal anesthesia significantly (310 out of 629, 44.8%) and moderately (198/692, 28.6%). When asked if procedures were canceled or postponed due to an opioid shortage, 549 out of 638 (86.1%) said no. This raises an interesting question in terms of how optimal analgesia or anesthesia was performed in the presence of an opioid shortage. In the author's experience, multimodal analgesic and anesthetic plans were typically put in place involving approaches such as infusions of ketamine, lidocaine, butorphanol, and/or dexmedetomidine and the use of regional anesthetic blocks (9, 32). It would be interesting to understand how veterinarians filled the gap left by the opioid shortage.

In terms of the potential for diversion of opioids, participants were more concerned about diversion of opioids from pets to clients (or presumably clients' friend or family) than to hospital staff. Only 171 out of 691 (24.7%) of participants were concerned about potential diversion of opioids to clinic staff. Although lower than the number concerned about diversion from pets to clients (441 out of 691, 63.5%), these results suggest that veterinarians are aware that individuals within their clinics are just as susceptible to opioid addiction as the rest of the population. It is not surprising that veterinarians would have a greater level of trust for people they work with on a daily basis as compared to some of their clients in terms of the potential for diversion.

Limitations of this study are that the data was gathered via an on-line survey and it is likely that those veterinarians which were impacted by the opioid shortages are probably more likely to complete the survey. In addition, the survey was administered to VIN members and not the entire population of veterinarians. A further limitation is that the survey was geared toward general practitioners so that we do not know the impact of the shortages on specialty practices.

The results of this study suggest that veterinarians have been impacted by the opioid shortage, with a greater impact on full mu opioid agonists as compared to drugs like butorphanol, buprenorphine, and tramadol. The shortage has had a moderate to significant impact on the delivery of analgesic care and optimal anesthesia to veterinary patients. In terms of dispensing or prescribing opioids, the impact of the opioid shortage appears to have been greater on the ability of the veterinarian to have the drugs available to dispense from their own pharmacy. Importantly, these results confirm the important role of opioid analgesics in the delivery of modern veterinary medicine in spite of significant societal problems associated with the overuse of opioids. This is essentially the same as in human health whereby opioids have an important and legitimate role in pain management and anesthesia, and highlights the importance of medical health professionals being able to access these critical medications (33).

## Data Availability

The datasets generated for this study are available on request to the corresponding author.

## Author Contributions

LK, PH, and MR conceived the study and conducted the research. RS-T helped write and prepare the manuscript.

### Conflict of Interest Statement

The authors declare that the research was conducted in the absence of any commercial or financial relationships that could be construed as a potential conflict of interest.
